# An atrial fibrillation rotor, mapped conventionally

**DOI:** 10.1111/jce.14329

**Published:** 2020-01-13

**Authors:** Niraj Varma, Raymond Rizzo, Brian Wisnoskey

**Affiliations:** ^1^ Department of Cardiac Electrophysiology, Heart and Vascular Institute Cleveland Clinic Cleveland Ohio

**Keywords:** atrial fibrillation, electroanatomic mapping, entrainment, reentry, rotor

## Abstract

This case illustrates that the condition of atrial fibrillation (AF) may harbor site(s) of regular rotational activity, reentry may be an underlying mechanism, high periodicity and wavebreak through areas of the scar may generate fibrillatory conduction, and disintegration of the “rotor” may not abolish AF.

A 67‐year‐old woman presented with palpitations and an irregular pulse, despite amiodarone therapy and pulmonary vein isolation for atrial fibrillation (AF) 1 year previously. Surface electrocardiogram indicated AF. Mapping demonstrated disorganized activity in the right atrium and left atrial body and confirmed pulmonary vein isolation. However, a localized (<20‐mm diameter) left atrial (LA) area showed regular electrical periodicity (185 ms) (Figure 1 and Supporting Information Video).

**Figure 1:**
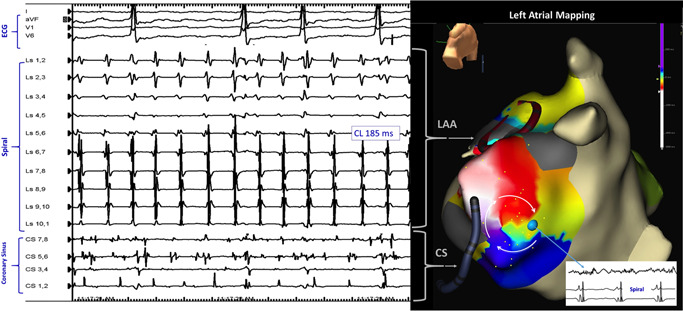
Left panel shows electrocardiogram leads (top, showing atrial fibrillation) and intracardiac electrograms recorded from a catheter in the left atrial appendage (spiral; LAA) and the coronary sinus (CS). Rhythm is regular in spiral (cycle length 185 ms, seen well in bottom 5 bipoles) but irregular in CS. Right panel shows electroanatomic LA map (left posterior oblique caudal) with electrode catheter positions. Point by point mapping revealed an area of rotational activity (clockwise arrows) covering the tachycardia cycle length (gradation of colors, total 37 points on this region) implying a reentrant circuit. A long fractionated electrogram was recorded at one point (blue dot, top channel in the inset, timed to 2 spiral electrograms below) suggestive of a zone of slow conduction. The rotor is bounded by dense scar areas (gray)

High frequency localized singularities (“rotors”) have been proposed as ablation targets for persistent AF.^1^ Identification relies usually on phase mapping of simultaneously acquired unipolar signals from multielectrode catheters (with limitations inherent to algorithmic assumptions). Here, we used classical clinical electrophysiology mapping techniques since the rotor anchored for several minutes and demonstrated a reentrant mechanism (Figure 2). (However, not all rotors may be reentrant). A reentrant circuit existing in the presence of fibrillation in the remainder of the chamber requires special conditions. Scar likely produce substrate for reentry, but also partial insulation of the circuit from afferent electrical waves.^2^ At the same time, scar presents conduction barriers to electrical activity propagating efferently from the rotor, to cause wavebreak and fibrillatory activity throughout the rest of the chamber (Supporting Information Video).

**Figure 2:**
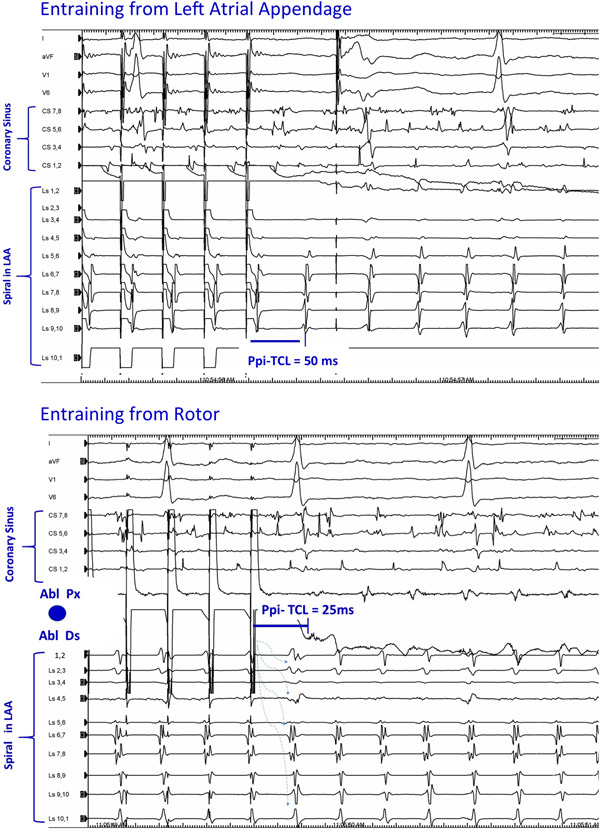
Entrainment (paced cycle length 170 ms) (top) pacing from the left atrial appendage (LAA) captured the regular electrograms inscribed on this spiral catheter (but coronary sinus [CS] was unaffected). This supports local reentry. Postpacing interval (PPi) was longer than the tachycardia cycle length (TCL) indicating that this region was not in the circuit. (Bottom) Pacing the region with fractionated electrograms (blue dot; Figure 1) with a roving catheter captured the regular electrograms on this catheter and the spiral catheter (but not the irregular activity on the CS catheter). The PPi‐TCL is 25 ms indicates the position within the reentrant circuit. Note that the first electrograms recorded on the spiral catheter following the last pacing artifact are driven at paced cycle length ie these are orthodromically driven through an area of slow and/or long conduction path (dashed arrows)

In this case, radiofrequency ablation to the circuit area disintegrated rotational activity but did not terminate AF. This may be because other rotors were not mapped, and/or that some rotational activity observed in the LA body (eg, those controlling small areas^3^) may not be critical to the maintenance of AF. These factors may contribute to the limited clinical success observed with current ablation strategies targeting rotors.

## Supporting information

Supporting information

Supporting information
