# Supplementary material for: An atrial fibrillation rotor, mapped conventionally
Source: J Cardiovasc Electrophysiol. 2020 Jan 13;31(2):544–6. doi: 10.1111/jce.14329 (PMC9292734; doi:10.1111/jce.14329)
Supplement: Supplementary file 2 — Supporting information [file JCE-31-544-s002.docx]

**Video Left atrial propagation map**

Wavefronts are shown in white and scar areas in grey. **Left panel** depicts left posterior oblique caudal view with CS catheter poles 2-8 and spiral catheter in the LAA. This shows high speed rotational activity with wavebreak at the boundary scar areas leading to fibrillatory conduction beyond. **Right panel** shows rotation with the zone of slow conduction corresponding to the zone demonstrating fractionated electrograms (blue dot, Figure 2 right panel).
